# Efficient Delivery of Antimicrobial Peptides in an Innovative, Slow-Release Pharmacological Formulation

**DOI:** 10.3390/pharmaceutics15112632

**Published:** 2023-11-16

**Authors:** Naroa Serna, Hèctor López-Laguna, Patricia Aceituno, Mauricio Rojas-Peña, Eloi Parladé, Eric Voltà-Durán, Carlos Martínez-Torró, Julieta M. Sánchez, Angela Di Somma, Jose Vicente Carratalá, Andrea L. Livieri, Neus Ferrer-Miralles, Esther Vázquez, Ugutz Unzueta, Nerea Roher, Antonio Villaverde

**Affiliations:** 1 Institut de Biotecnologia i de Biomedicina (IBB), Universitat Autònoma de Barcelona, 08193 Barcelona, Spain; naroas@nanoligent.com (N.S.); patriciaeliana.aceituno@autonoma.cat (P.A.); mauricioeduardo.rojas@uab.cat (M.R.-P.); eloi.parlade@uab.cat (E.P.); eric.volta@uab.cat (E.V.-D.); carlos.martinez.torro@uab.cat (C.M.-T.); julieta.sanchez@uab.cat (J.M.S.); angela.disomma@unina.it (A.D.S.); josevicente.carratala@uab.cat (J.V.C.); alivieri555@gmail.com (A.L.L.); neus.ferrer@uab.cat (N.F.-M.); esther.vazquez@uab.cat (E.V.); nerea.roher@uab.cat (N.R.); 2 Departament de Genètica i de Microbiologia, Universitat Autònoma de Barcelona, 08193 Barcelona, Spain; uunzueta@santpau.cat; 3 Centro de Investigación Biomédica en Red de Bioingeniería, Biomateriales y Nanomedicina, Instituto de Salud Carlos III, 28029 Barcelona, Spain; 4 Departament de Biologia Cel·lular, Fisiologia Animal i Immunologia, Universitat Autònoma de Barcelona, 08193 Barcelona, Spain; 5 Instituto de Investigaciones Biológicas y Tecnológicas (IIBYT), (CONICET-Universidad Nacional de Córdoba), ICTA, FCEFyN, UNC. Av. Velez Sarsfield 1611, Córdoba X 5016GCA, Argentina; 6 Biomedical Research Institute Sant Pau (IIB Sant Pau), 08041 Barcelona, Spain

**Keywords:** recombinant proteins, drug delivery, antimicrobial peptide, secretory granules, microparticles

## Abstract

Both nanostructure and multivalency enhance the biological activities of antimicrobial peptides (AMPs), whose mechanism of action is cooperative. In addition, the efficacy of a particular AMP should benefit from a steady concentration at the local place of action and, therefore, from a slow release after a dynamic repository. In the context of emerging multi-resistant bacterial infections and the urgent need for novel and effective antimicrobial drugs, we tested these concepts through the engineering of four AMPs into supramolecular complexes as pharmacological entities. For that purpose, GWH1, T22, Pt5, and PaD, produced as GFP or human nidogen-based His-tagged fusion proteins, were engineered as self-assembling oligomeric nanoparticles ranging from 10 to 70 nm and further packaged into nanoparticle-leaking submicron granules. Since these materials slowly release functional nanoparticles during their time-sustained unpacking, they are suitable for use as drug depots in vivo. In this context, a particular AMP version (GWH1-NIDO-H6) was selected for in vivo validation in a zebrafish model of a complex bacterial infection. The GWH1-NIDO-H6-secreting protein granules are protective in zebrafish against infection by the multi-resistant bacterium *Stenotrophomonas maltophilia*, proving the potential of innovative formulations based on nanostructured and slowly released recombinant AMPs in the fight against bacterial infections.

## 1. Introduction

Infectious diseases represent an increasing health threat, expected to achieve immense proportions in the next decades. The abuse and misuse of antibiotics have contributed towards the accumulation of drug-resistant genes in single bacterial strains that allow them to escape the activity of diverse sets of antibiotics [1]. These multidrug-resistant pathogens are responsible for high-impact, life-threating infections whose incidence is expected to increase in the forthcoming decades [2,3,4,5]. Beyond conventional antibiotics, unconventional antimicrobial (AM) agents are either identified from nature [6,7,8] and/or further developed in the laboratory through diverse engineering approaches [9,10,11]. This process is aimed at enhancing their effectiveness and offering a new spectrum of clinically realistic alternatives and formulations usable as operative AM drugs [12,13]. AMPs are among such new agents [14,15,16], usually produced via chemical synthesis but also suited for recombinant production in microbial cells via fusion to carrier proteins [17,18] or through tandem arrangements [19,20,21]. Of course, the recombinant biofabrication of AMPs in prokaryotic cell factories offers, as in the case of any other protein drug [22,23], fully scalable, eco-friendly, and cost-effective methodologies [24,25,26,27] compatible with a sustainable circular economy [27]. Also, recombinant AMPs benefit from the generic properties of protein drugs, namely, excellent biocompatibility and biodegradability and the possibility of functional and structural adjustment through conventional genetic engineering [28,29]. Since protein drugs are broadly used in human clinics [22,23], the possibility of developing recombinant AM pharmaceuticals is highly promising as the main strategic biochemical approach.

In the global effort to improve the effectiveness of AMPs, two main conclusions have been drawn. First, AMP presentation as nanoscale versions ensures particularly good tissue penetrability, stability, and retention in contrast to those of monomeric soluble versions, with multivalence and local cooperativity (in homomeric AMP oligomers) being highly desirable parameters [30,31,32,33]. Furthermore, as in the case of many other drugs, time-sustained AMP release might enhance effectiveness, prompting the development of well-suited materials, devices, and holding matrices [34,35,36]. In this context, we have previously generated recombinant versions of distinct AMPs that, in the form of self-assembling fusion proteins, can be easily produced in *Escherichia coli* as multivalent nanoscale oligomers [21,31,37] on which the displayed AMP is fully functional. On the other hand, we have recently developed a protein-only type of submicron/micron material [38] in which monomeric or oligomeric protein versions are clustered by the coordination between histidine residues of hexahistidine tags (H6) and divalent cations (such as Zn^2+^ or Ca^2+^) [38,39,40,41]. These ions act as a molecular glue between overhanging histidine residues from different polypeptides [42,43]. Then, the AMPs are self-contained within microparticles that, in the absence of any holding matrices, slowly disintegrate under physiological conditions via the spontaneous chelation of cations. This characteristic, which mimics the release of peptidic hormones from the human endocrine system [44,45,46,47], results in a steady leakage of the building block protein in cell culture [48] or in vivo [39]. The present study was designed to evaluate the AMP performance, in vitro and in vivo, as a formulation that combines these concepts, namely, recombinant AMP versions, organized as multimeric nanoparticles, which are slowly delivered from protein-only dynamic depots. The presented data fully support the usability and clinical potential of recombinant AMPs administered in such a novel protein-only depot formulation based on a very simple synthesis process.

## 2. Materials and Methods

### 2.1. Genetic Design, Production, and Purification

The codon-optimized genes encoding for AMP-based peptides, namely, T22, PaD, GWH1, and Pt5, along with a hexahistidine tag sequence, were devised and integrated into the pET22b vector via NdeI and HindIII restriction enzymes. Green fluorescent protein (GFP) and an engineered segment of human nidogen (NIDO [49]) were selected as protein scaffolds. The resulting plasmids were procured from GeneArt (ThermoFisher, Waltham, MA, USA), and the encoded proteins were subsequently produced in *Escherichia coli* and purified as previously reported [50], including a cell disruption step of three rounds of disruption (Emulsiflex-C5—Homogenizer; Avestin) at 500–1000 psi for recombinant protein extraction.

### 2.2. Purity, Concentration, and Integrity

**Figure 1:**
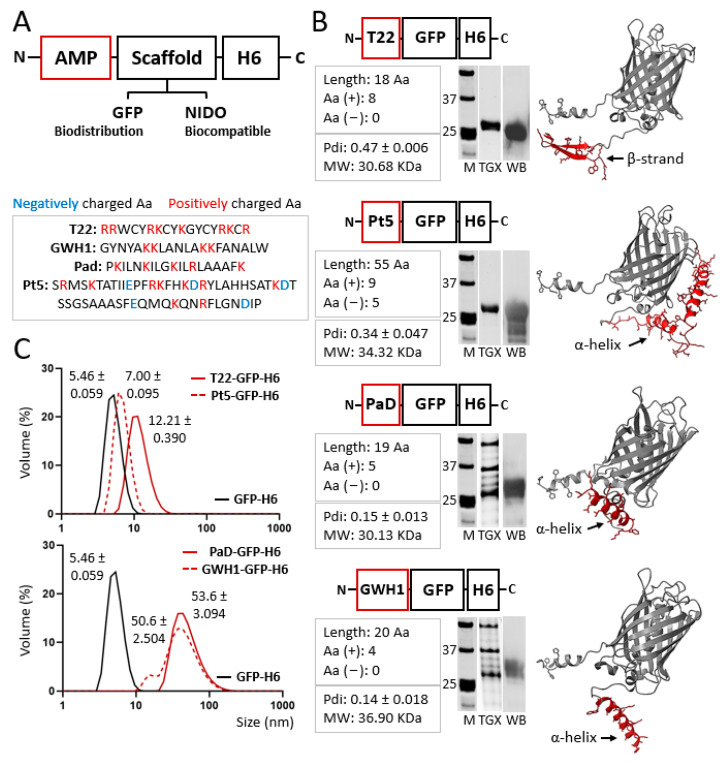
Physicochemical characterization of AMP-based recombinant proteins. (**A**) Top: Schematic representation of the modular design of the protein-based platform. The GFP scaffold is intended to be used as a reporter in biodistribution. NIDO, being a human protein segment, is a promising scaffold envisaging clinical uses. Bottom: AMP amino acid sequences. (**B**) Schematic modular representation of each protein construction based on an N-terminal AMP (namely, T22, Pt5, PaD, and GWH1). Right: The 3D structure prediction of each protein (AMP sequence displayed in red). Center: Protein integrity and purity analyzed using SDS-PAGE electrophoresis (TGX) and Western blot (WB; monoclonal anti-His antibody, Santa Cruz Biotechnology, Santa Cruz, CA, USA), respectively. Marker sizes are displayed between 37 and 25 KDa. Left: A short primary sequence description of each AMP, indicating peptide length and the charge of charged amino acids. The polydispersity index (Pdi) and the experimental molecular weight (MW) are also displayed underneath the description box. (**C**) Volume size distribution (in nm) of the resulting nanoparticles. Peak values are displayed ± standard error of the mean. The parental construct GFP-H6, which, under the used conditions, does not assemble into nanoparticles, is also displayed in black as a reference. The polydispersity index (Pdi) for each material is also displayed underneath the description box, in panel B, as mean ± standard error of the mean.

The physicochemical analyses of the proteins were conducted as previously reported [50], and the primary sequences of the AMP constructs (Figure 1A,B) were obtained using ProtParam online software (ExPASy, *Protein Identification and Analysis Tools on the Expasy Server*; https://doi.org/10.1385/1-59259-890-0:571, accessed on 6 November 2023). Protein integrity was assessed using matrix-assisted laser desorption ionization time-of-flight (MALDI-TOF) mass spectrometry.

### 2.3. Size, Ultrastructural Morphometry and Surface Charge Characterization

The volume size distribution (in nm) and ζ-potential (in mV) of the protein samples were evaluated using dynamic light scattering (DLS) and electrophoretic light scattering (ELS, Hongkong, China), respectively, at a wavelength of 633 nm and a standard temperature of 25 °C, employing a Zetasizer Advance Pro instrument (Malvern Instruments, Malvern, UK). Measurements for secretory granules were performed in 0.839 s as the measurement time. The ultrastructural morphometry of secretory granules deposited on 3M^TM^ Aluminum Conductive Tape (TED PELLA, Inc., Redding, CA, USA) was analyzed by Scanning Electron Microscopy (FESEM, Huntington Beach, CA, USA) without coating using a FESEM Zeiss Merlin (Zeiss, Oberkochen, Germany) operating at 2 kV and equipped with a high-resolution secondary electron detector.

### 2.4. Synthesis of Secretory Granules

The pure and soluble protein was initially adjusted to 2 mg/mL and subsequently aliquoted into fixed final volumes of 250 μL. Next, a filtered chloride solution of each divalent cation (i.e., Zn^2+^, Fe^2+^ or Mn^2+^, stock at 400 mM) was added to each tube, yielding precisely defined final concentrations of 8, 5, and 4 mM, respectively. The resulting mixtures were then gently mixed, incubated for 10 min at room temperature, and subjected to centrifugation for 5 min at 10,000× *g* to isolate the soluble and insoluble fractions for further analysis. The remaining protein in the soluble fraction was quantified by means of the Bradford assay, thereby allowing for the accurate estimation of the percentage of microstructured protein. Finally, the obtained protein pellets (i.e., secretory granules) were carefully stored at −80 °C until further use.

### 2.5. Chemical Detection of Zinc within Secretory Granules

The chemical detection of zinc in the secretory granules was conducted as previously reported [51], and results are expressed as μg of cation per mg of protein.

### 2.6. Protein Release from Secretory Granules

Stored secretory granules were thawed and gently resuspended in 500 μL of E3 0.1% Methylene Blue zebrafish medium (E3 medium) at room temperature. Particle suspensions were then incubated at 37 °C without agitation, and 75 μL aliquots were collected at several time points (0 h, 3 h, 24 h, 72 h, 120 h, and 168 h). Following centrifugation at 15,000× *g* for 15 min, soluble and insoluble fractions were separated, and the mobility and integrity of proteins determined using SDS-PAGE using a TGX Stain-Free FastCast Acrylamide Kit, 12% (BioRad, Hercules, CA, USA). Protein bands were additionally immunodetected via Western blot (WB) using a monoclonal anti-His antibody (Santa Cruz Biotechnology, Dallas, TX, USA), and their intrinsic intensity was quantified with Image Lab 5.2.1 software, allowing the calculation of the percentage of released protein.

### 2.7. In Vitro Bactericidal Assay in Staphylococcus aureus

The antimicrobial efficacy of diverse secretory granules against *S. aureus* ATCC-29737 was evaluated utilizing a broth microdilution method. In 96-well plates, antimicrobial samples were subjected to a 2-fold serial dilution process in Mueller Hinton Broth Cation-adjusted medium (MHB-2, Sigma-Aldrich, St. Louis, MI, USA), with each well containing a specific amount of the corresponding protein in the range of 0.5 to 8 μmol/L in 50 μL. Subsequently, 50 μL of MHB-2 medium with 10^6^ colony-forming units per mL (CFU/mL) of *S. aureus* was inoculated into each well. Plates were then incubated without agitation at 37 °C for a period of 18 h with 50 μg of secretory granules, and the number of colonies was counted and is expressed as log CFU/mL.

### 2.8. Zebrafish Husbandry and Breeding

Adult wild-type zebrafish (*Danio rerio*) were maintained in a recirculating aquarium at a constant water temperature of 28 ± 1 °C under a 12 h light:12 h darkness photoperiod. Fish were fed twice daily at 2% body weight. Water parameters were monitored weekly, with pH maintained at 6.8–7.5; ammonia and nitrite levels were kept below detection limits; and nitrate levels were maintained below 100 mg/L. Breeding was induced by transferring 2 females and 4 males to a separate tank, and fertilized eggs were collected the following morning and cultured in E3 medium in Petri dishes. All animal experiments were conducted following EU 2010/63 guidelines and approved by the ethics committees at Universitat Autònoma de Barcelona (UAB, CEEH, Bellaterra, Spain).

### 2.9. In Vivo Biodistribution in Zebrafish Larvae

Groups of five larvae (*n* = 5 per condition, 72 h post-fertilization) were distributed on 6-well plates (Thermo Fisher Scientific) and immersed in 1 mL of E3 medium (control), or 50 µg/mL of T22, PaD, Pt5 and GWH1 AMP-based granules, and soluble GFP-H6. Biodistribution after 48 h treatment was observed in anesthetized larvae (50 ppm, MS-222) using a fluorescence stereomicroscope (Nikon SMZ800, Tokyo, Japan) coupled with a camera (Nikon DS-Fi2).

### 2.10. In Vivo Model of Infection: Stenotrophomonas maltophilia (K279 Strain) in Adult Zebrafish

Zebrafish (*D. rerio*), averaging 45 mm in size and 0.8 g in weight, were acclimated in 5 L tanks at 28 °C for 2 days before treatment. Two infection trials were conducted: In the first trial, zebrafish were divided into eight groups (*n* = 12), including a control group (PBS), Zn^2+^, PBS containing 25 or 50 µg of artificial GWH1-NIDO-H6 SG without infection to test for toxicity, and PBS containing 25 or 50 µg of each nano- or micro-material injected intraperitoneally and co-administered with K279 at 1.8 × 10^9^ CFUs/mL. In the second trial, zebrafish were divided into three groups (*n* ≥ 10), including a control group, and GWH1-NIDO-H6 SG 50 µg was administered 24 h before infection by intraperitoneal injection with K279 at 1.6 × 109 CFU/mL. In both experiments, a K279 group was included as a negative survival control. The mortality rate of each group was recorded daily.

### 2.11. 3D Modelling

The three-dimensional (3D) conformations of proteins’ folded state were computationally predicted using the AlphaFold2 [52] algorithm. The algorithm constructs a multiple sequence alignment (MSA) by finding similar sequences to the input (e.g., sequences identified in living organisms and proteins with similar structures) and extracts the most representative information using a particular neural network architecture that passes that information through to a second neural network that produces a structure. This procedure is iteratively repeated to refine the final structural prediction. What is more, this algorithm is integrated into the ColabFold [53] platform, and the default software settings were utilized. Each primary FASTA sequence was introduced as a query individually for prediction, and ChimeraX 1.3 software was run for 3D structure processing and visualization, in which the AMPs’ 3D structures were highlighted in red.

### 2.12. Statistical Methods

To confirm the normal distribution of data, preliminary normality and log-normality tests, namely, Anderson–Darling, D’Agostino and Pearson, Shapiro–Wilk, and Kolmogorov–Smirnov, were conducted. For parametric data, one-way or two-way analysis of variance (ANOVA) or *t*-tests were applied, depending on the number of groups and conditions. On the other hand, nonparametric data were analyzed using the Kruskal–Wallis test. All measurements were performed at least in triplicate, and the peak values are expressed as mean ± standard error (SE) using GraphPad Prism 8 software. Significance was established at (*) *p* < 0.05.

## 3. Results

Four AMPs, namely, T22 [37], GWH1 [54], PaD [55], and Pt5 [56], were synthesized to be displayed on nanoscale oligomers through N-terminal fusions to a H6-tagged GFP (Figure 1A,B). The cationic characteristic of these peptides favors cation-assisted oligomerization through cross-molecular contacts that involve histidine residues from the H6 tails [51]. All proteins were produced in good yields in *E. coli* in the form of full-length fusions, with expected electrophoretic mobility and molecular weights (Figure 1B). Also, they spontaneously assembled upon purification from bacterial cell extracts as nanoparticles of different sizes (Figure 1C). The biomolecular basis for oligomerization is the combination of an N-terminal cationic peptide and a C-terminal histidine-rich peptide, with the intervention of divalent cations [42]. Under the tested conditions, the parental construct GFP-H6, used here as a negative control, did not assemble (Figure 1C).

**Figure 2:**
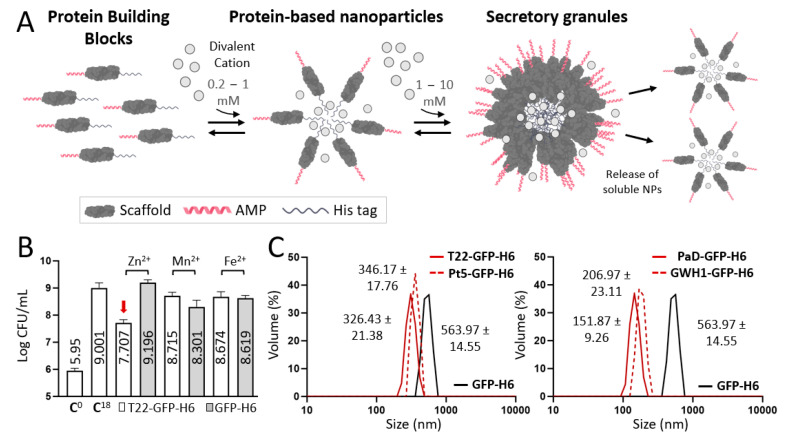

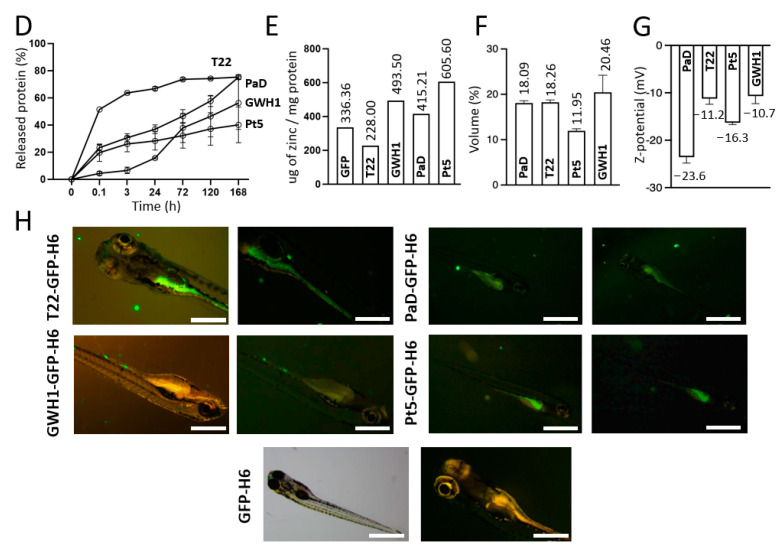
Manufacturing of AMP-based secretory granules (SGs) and in vivo biodistribution. (**A**) Schematic representation of the formulation process upon divalent cation addition (from recombinant proteins acting as building blocks for artificially created SGs, which release functional protein). Ranges of divalent cations are displayed on top of the right-pointing arrows. Scaffold is displayed in dark grey, AMP structure in red, and histidine tag in pale grey. (**B**) *S. aureus* bacterial growth (log CFU/mL) after 18 h in presence of T22-GFP-H6 (white bars) or GFP-H6 (grey bars) in artificial SGs constructed with different divalent cations (namely, zinc II, manganese II, and iron II). C^0^ refers to the amount of initial bacterial colonies, and C^18^ after 18 h of incubation. Numbers inside the bars refer to log CFU/mL for each material. Red arrow displays the most effective condition. (**C**) Volume size distribution analysis (nm) by DLS of each zinc-based artificial SG. Peak values are displayed ± standard error of the mean. A positive control of protein microparticularization, namely, GFP-H6, is also displayed in black. (**D**) Protein release (in percentage) at 37 °C from each zinc-based artificial SG upon time (from 0 to 168 h). (**E**) Chemical detection of zinc II (in µg) for each artificial SG. Peak values refer to the number of ug per mg of protein for each condition. (**F**) Volume-size distribution analysis (in nm; peak values displayed on top) via DLS of released soluble protein from each zinc-based artificial SG following a 24 h incubation at 37 °C. (**G**) Z-potential analysis (in mV; peak values displayed on top) of released soluble protein from panel F. (**H**) In vivo biodistribution of zinc-based artificial SGs in zebrafish larvae at 50 µg/mL for 48 h. White bar represents 500 µm.

Using these nanoparticles as starting components, the synthesis of higher-order microscale particles was approached by testing alternative divalent cations as cross-molecular linkers (Figure 2A). In this regard, chloride salt versions of Zn^2+^, Fe^2+^, and Mn^2+^ were tested in a molar excess to act as a supply of divalent cations for protein clustering, using T22-GFP-H6 [37] as an indicator of AM activity (Figure 2B). All these cations were able, with variable efficacies, to promote protein microparticulation because of the overhanging H6 tags in them [43]. Because of the robustness of Zn^2+^ as a crosslinking agent [42] and since the materials formed with Zn^2+^ showed a clearer AM activity (Figure 2B), this cation was selected for further synthesis of protein granules out of the soluble nanoparticles. It must also be noted that Zn^2+^ ensures a good biocompatibility of the materials since its concentration in a conventional dosage of microparticles, translated to humans, falls within the daily recommended allowance [42], in contrast to other alternative divalent metals. Also, among the divalent cations that we have previously tested for the fabrication of microparticles, Zn^2+^ allows a much smoother and prolonged release of the protein building blocks [39,57].

As observed in Figure 2C, under the tested conditions, the newly formed supramolecular complexes of all the constructs occurred at the submicron scale, between 150 and 600 nm, exhibiting amorphous morphologies (Supplementary Figure S1). Protein release was monitored from these materials over time and in physiological buffer, revealing protein-dependent variability in this process (Figure 2D). This was indicative of the differences in the compactness and leakage availability of the nanostructured AMPs (ranging from 11 to 21 nm, Figure 2F). These agents exhibited a negative net surface charge (−10 to −24 mV) in all protein-based assemblies (Figure 2G), which might influence and modulate final clinical applicability. While the T22-based material was released very rapidly, PaD and especially GWH1 rendered more progressive release patterns. The different amounts of Zn^2+^ retained by the particles during formation (Figure 2E) did not offer clues for the differential release kinetics observed in Figure 2D.

To determine the potential toxicities of these materials and to evaluate their distribution in vivo, we employed a simple zebrafish larvae model that has been fully validated for toxicology [58,59]. In this context, the exposure of zebrafish larvae to 50 µg/mL of all versions of AMP-based granules for 48 h did not result in any sign of toxicity. Also, after 48 h of immersion, the materials derived from T22, PaD, and Pt5 were found in the gastrointestinal tract and pancreas of the zebrafish larvae, whereas the fluorescence of GWH1-based granules was only observed in the intestine. Interestingly, in larvae treated with soluble GFP-H6, no fluorescent signal was observed (Figure 2H). Although the biological reasons for this biodistribution should be further explored, in case it might be clinically relevant, these data indicate that the oligomers remain in such a state in vivo, since retention effects are clearly observed in the assembled materials but not in the soluble protein. This is supportive of the structural robustness of the protein assembling platform used here and of the powerful protein–protein interactivity principles that underlie the resulting materials. To further move towards a functional analysis, the same in vivo model was used to determine the AM properties of these materials. In particular, the granules formed by GWH1 were the ones that rendered a more constant and steady protein release (Figure 2D) and showed a more localized biodistribution tendency. Therefore, they were deemed interesting for further analyses.

**Figure 3:**
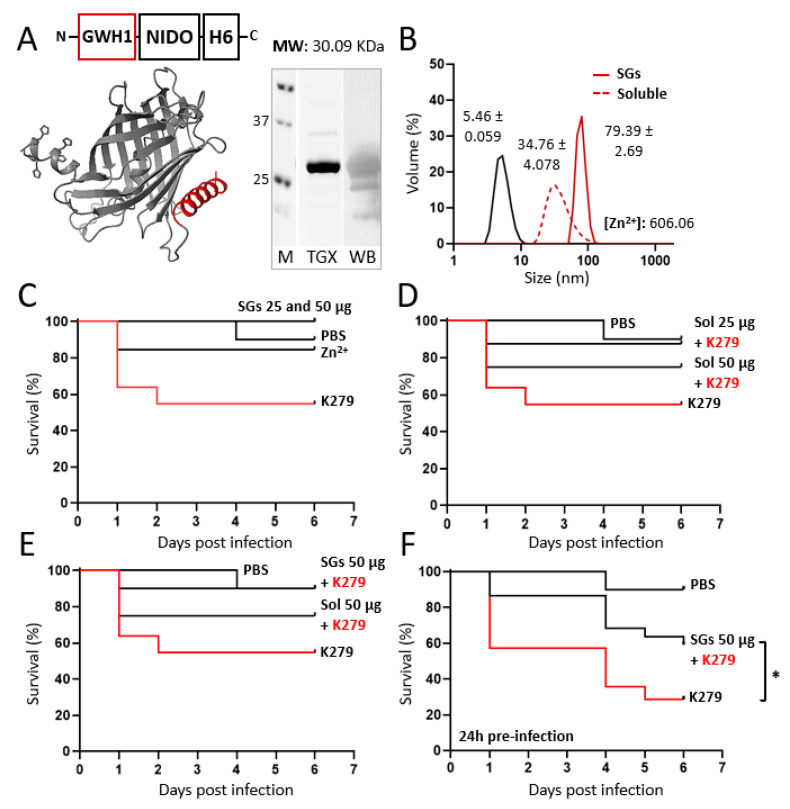
Humanizing AMP-based SGs, physicochemical characterization, and in vivo AM effects. (**A**) Schematic modular representation of the protein-based construction. Right: Integrity, experimental molecular weight (MW), and purity analyzed using SDS-PAGE electrophoresis (TGX) and Western blot (WB), respectively. Marker sizes are displayed in between 37 and 25 KDa. Left: Three-dimensional structure prediction. AMP sequence displayed in red. (**B**) Volume size distribution analysis (nm) via DLS of both AMP-based nanoparticles and artificial SGs. Peak values are displayed as ± standard error of the mean. A negative control of protein assembling, namely, GFP-H6, is also displayed in black. The amount of chemically detected zinc II, [Zn^2+^], is also displayed in (µg/mg of protein). (**C**) Toxicity assay expressed as organism survival (in percentage) in adult zebrafish upon addition of 25–50 µg of artificial GWH1-NIDO-H6 SGs and free zinc until day 6 without infection. PBS is displayed as positive survival control and K279 (infective bacteria) as negative control. (**D**) Effective dose screening expressed as organism survival (in percentage) in adult zebrafish upon addition of 25 and 50 µg of soluble GWH1-NIDO-H6 nanoparticles until day 6 post-infection with K279. PBS is displayed as positive survival control and K279 as negative control. (**E**) Antimicrobial effect expressed as organism survival (in percentage) in adult zebrafish after the administration of 50 µg of both soluble nanoparticles and artificial SG of GWH1-NIDO-H6 until day 6 post-infection with K279. PBS is displayed as positive survival control and K279 as negative control. (**F**) Antimicrobial effect expressed as organism survival (in percentage) in adult zebrafish after the administration of 50 µg of artificial SG of GWH1-NIDO-H6 (administered 24 h pre-infection) until day 6 post-infection with K279. PBS is displayed as positive survival control and K279 as negative control. Statistical significance was achieved when *p* < 0.05 (*).

For that reason, a humanized version of the GWH1 fusion protein was generated, in which GFP was replaced by a human structural analogue (Figure 1A and Figure 3A), namely, the beta barrel domain of the human nidogen [60]. This protein segment was successfully used by us as a structural replacement for GFP, serving as a scaffold for nanoparticle formation from multidomain proteins, acting as drug carries in cancer, and it seemed adequate as a scaffold for peptide display in nanoscale oligomers [49]. The resulting GWH1-NIDO-H6 nanoparticles of around 34 nm formed, upon addition of cationic Zn^2+^, higher-order materials with final sizes comparable to those based on GFP (Figure 3B).

In non-infected larvae, neither the resulting granules nor the soluble Zn^2+^, at molar amounts equivalent to those present in the granules, showed any sign of toxicity (Figure 3C). When exposing the released soluble protein nanoparticles from secretory granules to larvae infected with *S. maltophilia* (strain K279), the AM activity of the construct was confirmed, as it reduced the rate of death promoted by the bacterial infection (Figure 3D). When using 50 µg of GWH1-NIDO-H6 in the form of secretory granules, the survival of the larvae was slightly higher (but not statistically significant) than when exposing the larvae to the plain soluble format of the construct (Figure 3E) at equal concentration. Interestingly, the AM activity of the GWH1-NIDO-H6 granules was still detectable upon their addition to the media 24 h before infection, also rendering an important reduction in the mortality of the infected larvae (Figure 3F).

## 4. Discussion

The results presented here demonstrate that the oligomeric assembly of AMP-containing modular H6-tagged proteins (Figure 1) does not prevent their biological activity (Figure 2 and Figure 3). This activity is retained even when these proteins form complex supramolecular materials at the submicron/micron scales and under complex media (Figure 3). In vitro, such materials allow the steady release of the forming protein in an oligomeric form (Figure 2A,D), through kinetic patterns that are distinguishable depending on the cation used for assembly as clusters. Ionic Zn^2+^, as gluing agent, promoted a regular protein release in vitro over the testing period (almost three days; Figure 2D), and it was, among the other trialed cations (Fe and Mg), the one able to create the most functional granules (Figure 2B). The slow release of drugs or antigens is an emerging strategy in innovative medicine, especially appealing for immune stimulation [61] and the treatment of chronic or prolonged conditions [62,63]. In this regard, developing biocompatible holding matrices is a main bottleneck [64,65]. The use of Zn^2+^ or related divalent cations as a protein clustering agent allows the construction of microscale materials [38], which, through their slow physiological disintegration, release the forming building block protein [39], that is, the drug itself. Such a mechanism is found in nature and is used by peptidic hormones in their body storage through secretory granules, mostly based on Zn^2+^ [45,46,47]. The absence of chemically heterogeneous holding materials represents an advantage over analogous systems. We previously tested this principle in oncology, always under physiological conditions, namely, through subcutaneous injection [38]. The secretory granules, then, had never been tested for stability and performance out of the body under non-physiological conditions like the water in fisheries. The plain nanoparticle version and the granular secretory versions, in the aquatic media inherent to the in vivo model tested here, are similar in their capacity to protect zebrafish larvae from infection (Figure 3E). However, the tendency towards a stronger effect in the granular version (Figure 3E), combined with the stability of the granules in complex media (Figure 3F), allows envisaging AMPs in such clustered formulation as valuable therapeutic agents. This concept should apply not only in aquaculture but also in other clinical settings in which a slow protein drug release and multivalence might be required, especially in complex media.

## 5. Conclusions

Clustering His-tagged AMPs, as protein-only secretory granules at the microscale, supported by Zn^2+^-His biochemical coordination, maintain the biological activity of such agents, and they allow a slow release of the forming materials in a nanostructured way. The secretion process is functional in vivo upon oral delivery via immersion in fish models, a fact that confirms the structural and functional robustness of the microscale materials. The conferred protection and enhanced survival in an infection model based on a multi-resistant bacterial species demonstrate the usefulness of the concept and its applicability to a wide spectrum of clinical settings, even those that might challenge the stability and performance of the material. Finally, the structural versatility of the scaffold agent used for the AMP display and presentation allows its humanization through human-derived protein segments, a fact that opens the spectrum of the envisaged applications of artificial protein-based secretory granules.

## Data Availability

The data is available using the following https://doi.org/10.34810/data913 (accessed on 6 November 2023).

## Supplementary Materials

The following supporting information can be downloaded at: https://www.mdpi.com/article/10.3390/pharmaceutics15112632/s1, Figure S1: Ultrastructural morphology of AMP-based secretory granules. White bars refer to 2 μm.
