# Supplementary material for: Efficient Delivery of Antimicrobial Peptides in an Innovative, Slow-Release Pharmacological Formulation
Source: Pharmaceutics. 2023 Nov 16;15(11):2632. doi: 10.3390/pharmaceutics15112632 (PMC10674355; doi:10.3390/pharmaceutics15112632)
Supplement: Supplementary file 1 [file pharmaceutics-15-02632-s001.zip › pharmaceutics-2697227-supplementary.pdf]

pharmaceutics

MDPI

Article

Efficient Delivery of Antimicrobial Peptides in an Innovative, Slow-Release Pharmacological Formulation

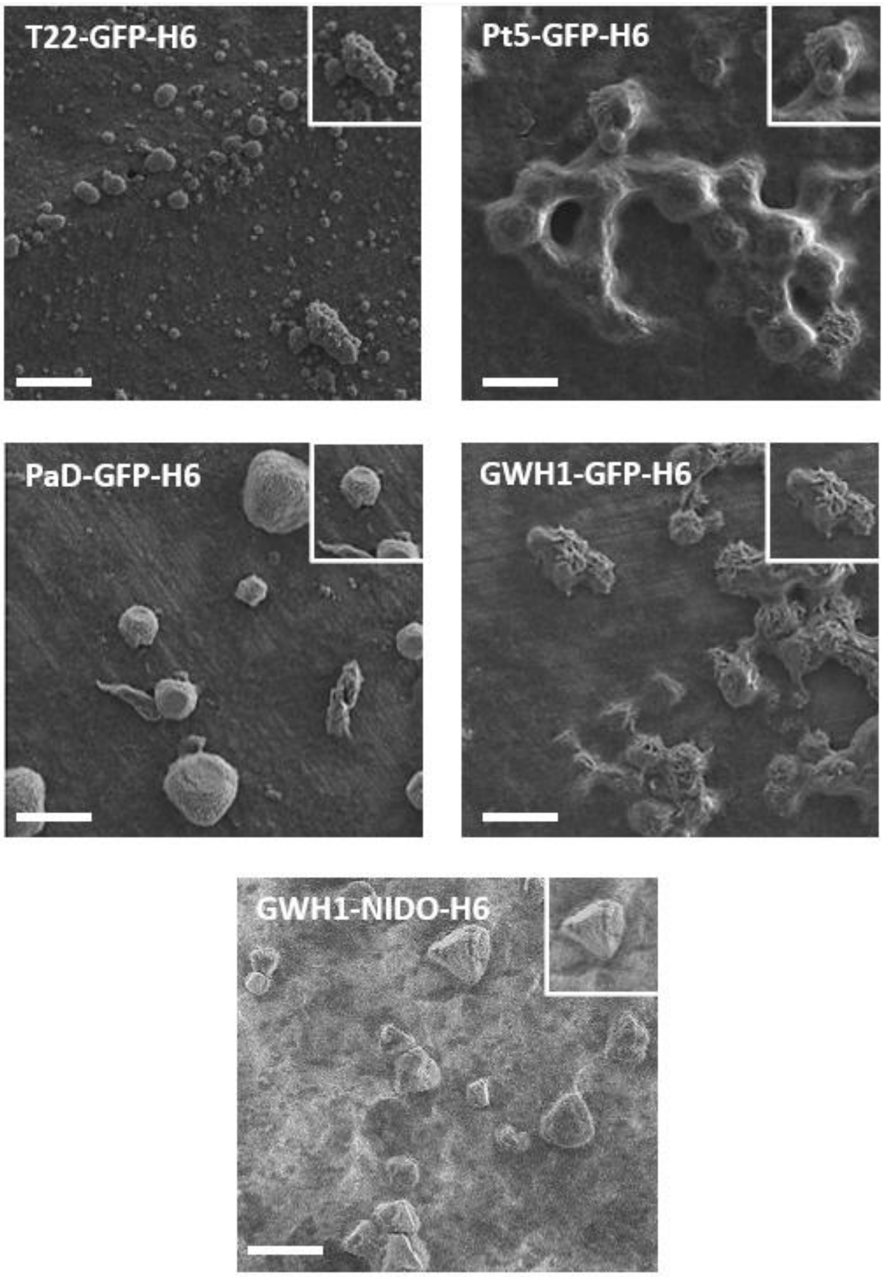

Supplementary Figure S1. Ultrastructural morphology of AMP-based secretory granules. White bars refer to 2 µm.
